# Draft genome sequence of *Staphylococcus saprophyticus* strain Nasal Sample 2 isolated from a milkman handling cows with subclinical mastitis in Kiruhura District, Uganda

**DOI:** 10.1128/mra.01180-25

**Published:** 2026-02-09

**Authors:** Reagan Muhwezi, Vidya Sankarapandian, G. R. Neel, Emmanuel Eilu, Ambrose Shabohurira, Ibrahim Ntulume, Lutaaya Festus, Priscilla Peter Dilli, Theophilus Pius, Naheem Adekilekun Tijani, Danladi Makeri

**Affiliations:** 1 Department of Microbiology and Immunology, Kampala International University Western Campus 365672 , Bushenyi, Western Uganda; 2 Institute of Allied Health Sciences, Clarke International University 127813, Kampala, Uganda; 3 College of Veterinary Medicine, Animal Resources and Biosecurity (CoVAB), Makerere University, Kampala, Uganda; 4 Department of Public Health, Kampala International University Western Campus 365672 , Ishaka, Uganda; 5 Department of Medical Laboratory Science, Kampala International University Western Campus 365672 , Ishaka, Uganda; University of Pittsburgh School of Medicine, Pittsburgh, Pennsylvania, USA

**Keywords:** subclinical mastitis, milkmen, *Staphylococcus aureus*

## Abstract

We report the draft genome sequence of *Staphylococcus saprophyticus* strain of Nasal Sample 2 (NS2) isolated from a milkman handling cows with subclinical mastitis in Kiruhura District, Uganda. The assembled genome comprises 2,773,281 bp with a guanine and cytosine (GC) content of 33%, 14 contigs, N50 (2,590,816 Mb), and 1 L50.

## ANNOUNCEMENT

Zoonosis is defined by the Centers for Disease Control and Prevention (CDC) as illnesses that people and animals can contract from one another (1). Subclinical mastitis (SCM) is among them that poses significant challenges in dairy farming (2). Due to its asymptomatic nature, it leads to economic losses and food insecurity in developing countries where access to advanced veterinary laboratory services is limited (3, 4). This data set provides genomic insights into resistance and virulence genes of *Staphylococcus saprophyticus* from a milkman handling cows with subclinical mastitis, offering valuable information for researchers studying antibiotic stewardship, disease evolution, and genetic surveillance.

Strain Nasal Sample 2 was isolated from a nasal sample collected from a milkman handling cows with subclinical mastitis in Kiruhura District, Uganda. The isolate was cultured on blood agar (Himedia, India) and incubated aerobically at 37°C for 24 h. Bright white, creamy spots, non-hemolytic (gamma-hemolytic) colonies indicative of *S. saprophyticus* presumptive isolates were purified further using blood agar, a differential media. Presumptive isolates were confirmed by positive catalase, negative coagulase, and urease. Antibiotic susceptibility testing of pure isolates was conducted using Kirby-Bauer standard disk diffusion method on Mueller-Hinton agar (MHA) in line with Clinical and Laboratory Standards Institute (CLSI) guidelines (5, 6). Penicillin (10 units; 6 mm), erythromycin (15 μg; 10 mm), tetracycline (30 μg; 12 mm), novobiocin (30 μg; 6 mm), and cefoxitin (30 μg, 18 mm) were among the few antibiotics to which the strain demonstrated resistance, particularly methicillin resistance.

The NS2 isolate was cultured in Luria-Bertani (LB) broth incubated in a shaker at 200 rpm for 18–24 h at 37°C, and DNA was extracted using column-based extraction using the NEB Monarch Spin gDNA Extraction Kit, including the extra lysozyme pre-treatment step for efficient lysis of gram-positive cells (7). DNA concentration and purity were also measured using a Qubit 3.0 fluorometer (Thermo Fisher Scientific) with the Quant-i­T dsDNA Assay Kit(Invitrogen), and NanoDrop ND-1000 spectrophotometer (Thermo Fisher Scientific). The MinION MK1B (MIN-101B) (Oxford Nanopore Technologies) platform was used for draft genome sequencing producing 1D reads (8, 9). Library preparation was conducted using the Rapid Barcoding Kit 96V14(SQK-RBK114.96) (Oxford Nanopore Technologies). An R10 flow cell (FLO-MIN114, Oxford Nanopore Technologies Lot#) was loaded with the prepared library (10).

The length of the assembled genome was 2,773,281 bp, 14 contigs, 2,590,816 N50, 2,590,816 N90, 1 L50, 1 L90, 33.07% GC, 91.3% depth coverage, and 8.7% minimum coverage. Raw reads were quality-checked with NanoPlot (v1.46.0) before (11) and after trimming. Adapter sequences and low-quality bases were trimmed using NanoFilt (v2.8.0) (11). All high-quality trimmed reads were used for *de novo* assembly with Flye (*v2.9.5-b180)* (12) and basecalling with (*SUP, v4.3.0*) (13) and polished with Medaka. All assembled reads passed the quality assessment using QUAST (v5.3.0) (13), and assembly completeness was determined using BUSCO (v5.8.3) (14). Resulting in 32,716.0 total reads, 173,747,489 total bases, 9,336 read length N50, and 24,494 (74.9%) 151.2 Mb > Q10 before trimming, after trimming had 29,064 total reads, 172,831,550 total bases, 9,435 read length N50, and 24,207 (83.3%) 151.0Mb > Q10. The assembled genome was submitted and annotated using the National Center for Biotechnology Information (NCBI) Prokaryotic Genome Annotation Pipeline (PGAP) (v6.10) (14). Quality analysis of the genome using CheckM analysis (v1.2.4) revealed 55.57% completeness and 8.83% contamination. Average nucleotide identity (ANI) analysis revealed that the closest type strain to our isolate was *Staphylococcus saprophyticus* (GCA_000010125.1) with an ANI of 99.57% and a genome coverage of 86.06%, confirming species-level identity and close relatedness, and yielded a genome length of 2,773,281 bp, 14 contigs with an N50 of 2,590,816, 1 L50, and a GC content of 33% after submission, as shared in Table 1.

**TABLE 1:** Data availability, genome statistics, and features of *Staphylococcus saprophyticus* NS2

Parameter	Value
Genome size	2,773,281 bp
Total ungapped length	2.8 Mb
Number of scaffolds	14
Scaffold N50	2.6 Mb
Scaffold L50	1
Number of contigs	14
Contig N50	2.6 Mb
Contig L50	1
GC percent	33
Genome coverage	63.3×
Assembly level	Contigs

## Data Availability

This draft Genome Shotgun study has been submitted to NCBI GenBank with accession number JBRLAS000000000.1 (15). Raw readings are accessible in the NCBI SRA database, accession number SRR35683872 (16). The accession number for the annotated genome is GCA_053037765.1 (17), and its BioSample is available under accession number SAMN52085326 (18).
